# Bifunctional Role of the Sternohyoideus Muscle During Suction Feeding in Striped Surfperch, *Embiotoca lateralis*

**DOI:** 10.1093/iob/obaa021

**Published:** 2020-07-29

**Authors:** J J Lomax, T F Martinson, Y E Jimenez, E L Brainerd

**Affiliations:** 1 Department of Ecology and Evolutionary Biology, Brown University, Providence, RI 02906; 2 Friday Harbor Labs, University of Washington, Friday Harbor, Washington, 98250

## Abstract

In ray-finned fishes, the sternohyoideus (SH) is among the largest muscles in the head region and, based on its size, can potentially contribute to the overall power required for suction feeding. However, the function of the SH varies interspecifically. In largemouth bass (*Micropterus salmoides*) and several clariid catfishes, the SH functions similarly to a stiff ligament. In these species, the SH remains isometric and transmitts power from the hypaxial musculature to the hyoid apparatus during suction feeding. Alternatively, the SH can shorten and contribute muscle power during suction feeding, a condition observed in the bluegill sunfish (*Lepomis macrochirus*) and one clariid catfish. An emerging hypothesis centers on SH muscle size as a predictor of function: in fishes with a large SH, the SH shortens during suction feeding, whereas in fish with a smaller SH, the muscle may remain isometric. Here, we studied striped surfperch (*Embiotoca lateralis*), a species in which the SH is relatively large at 8.8% of axial muscle mass compared with 4.0% for *L. macrochirus* and 1.7% for *M. salmoides*, to determine whether the SH shortens during suction feeding and is, therefore, bifunctional—both transmitting and generating power—or remains isometric and only transmits power. We measured skeletal kinematics of the neurocranium, urohyal, and cleithrum with Video Reconstruction of Moving Morphology, along with muscle strain and shortening velocity in the SH and epaxial muscles, using a new method of 3D external marker tracking. We found mean SH shortening during suction feeding strikes (*n* = 22 strikes from four individual *E. lateralis*) was 7.2 ± 0.55% (±SEM) of initial muscle length. Mean peak speed of shortening was 4.9 ± 0.65 lengths s^−1^, and maximum shortening speed occurred right around peak gape when peak power is generated in suction feeding. The cleithrum of *E. lateralis* retracts and depresses but the urohyal retracts and depresses even more, a strong indicator of a bifunctional SH capable of not only generating its own power but also transmitting hypaxial power to the hyoid. While power production in *E. lateralis* is still likely dominated by the axial musculature, since even the relatively large SH of *E. lateralis* is only 8.8% of axial muscle mass, the SH may contribute a meaningful amount of power given its continual shortening just prior to peak gape across all strikes. These results support the finding from other groups of fishes that a large SH muscle, relative to axial muscle mass, is likely to both generate and transmit power during suction feeding.

## Introduction

Suction feeding is a powerful and complex process of prey ingestion that relies on contributions from both head and body muscles in fishes. Whereas the cranial and hypobranchial muscles in the head region might intuitively be seen as the main drivers behind suction feeding, the axial muscles of the body have long been understood as necessary contributors to the process as well (Muller and Osse 1984; Westneat 1990; Van Wassenbergh et al. 2007). In many fishes, epaxial muscles contract to rotate and lift the neurocranium while the hypaxial muscles contribute to hyoid depression via pectoral girdle retraction (Lauder 1979, 1982; Liem 1980; Camp and Brainerd 2014).

The sternohyoideus (SH) muscle serves as a bridge between the body and the head, transmitting hypaxial muscle power to the hyobranchial elements of the head yet still retaining the potential to act on these elements itself. Recent studies have shown that axial musculature provides a majority of the power for strikes in largemouth bass (*Micropterus salmoides*) and bluegill sunfish (*Lepomis macrochirus*; Camp et al. 2015; Camp et al. 2018). In both species, the SH muscle is the only muscle in the head that is large enough to contribute more than negligible power. In largemouth bass, however, the SH function has been found to vary, as the muscle sometimes shortens and other times lengthens around the time of peak gape (Carroll 2004). Peak suction power production occurs just before peak gape, and the SH was later determined to show little to no shortening during periods of peak power production in *M. salmoides* (Camp et al. 2015). This lack of shortening during peak power indicates that the largemouth bass SH contributes little to no positive work and power. In contrast, bluegill sunfish rapidly shorten the SH during suction feeding, contributing power for both hyoid depression and, by extension, suction expansion (Camp et al. 2018). However, the contribution of the SH is still small, likely providing less than 10% of overall power, though the constant shortening of the bluegill sternohyoid for all studied strikes suggest that 10% may be crucial to generating a successful strike in the bluegill.

These muscle strain patterns indicate that in largemouth bass, the SH is active isometrically at the time of peak power production, effectively serving to transmit pectoral girdle rotation and hypaxial muscle power for hyoid depression (Carroll 2004; Carroll et al. 2004; Camp and Brainerd 2014; Camp et al. 2015), whereas in bluegill it serves a dual function both transmitting hypaxial power and contributing its own additional power for hyoid depression. In bluegill, the SH muscle is nearly twice the size of the SH in bass, when measured as a fraction of axial muscle mass (Camp et al. 2015; Camp et al. 2018). Clariid catfishes display this same relationship between SH muscle size and function. Of four species studied, the SH is largest in *Gymnallabes typus*, and this is the only species that showed SH shortening during suction feeding (Van Wassenbergh et al. 2007). In other studied clariid species, SH muscles were about half the size of *G. typus* and contracted isometrically during the first half of hyoid depression and then lengthened during the second half (Van Wassenbergh et al. 2007).

The clariid catfish and centrarchid results together suggest that the SH may be more likely to shorten and contribute power to suction expansion when the SH is relatively large, whereas, in species in which it is smaller, the SH does not shorten but instead acts much like a stiff ligament to transmit force and power from the hypaxial musculature to the hyoid. If muscle size is to be considered an indicator of sternohyoid bifunctionality, as both a transmitter and contributor to suction power, then studies focused on testing this prediction in species that fit this emerging size-specific criterion are necessary.

This study focuses on the suction feeding mechanism of the striped surfperch, *Embiotoca lateralis*, a species with a large SH muscle relative to *M. salmoides* and *L. macrochirus* (the mass of the SH is 8.8% of axial muscle mass in *E. lateralis* relative to 4.0% in *L. macrochirus* and 1.7% in *M. salmoides*; see “Materials and methods” and “Results” sections for more information on these masses). Does the surfperch SH shorten during suction expansion? We use a relatively new method, Video Reconstruction of Moving Morphology (VROMM) (Jimenez et al. 2018; Hoffmann et al. 2019) to track and animate the neurocranium, cleithrum, and a reference body plane, as well as a new method that employs external 3D marker tracking to estimate epaxial and SH muscle strain. As this particular application of external marker tracking is previously undescribed, we additionally present an experimental evaluation of the new method.

## Materials and methods

**Fig. 1:**
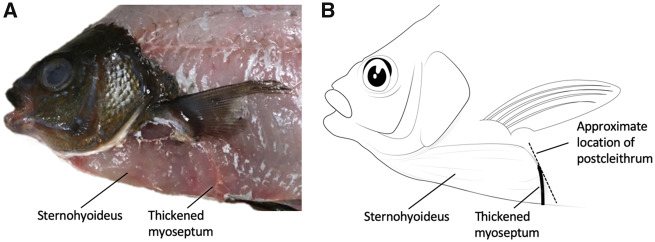
Anatomy of the SH muscle in the striped surfperch (*E. lateralis*) in lateral view. (**A**) Photograph of superficial dissection of a fresh specimen. (**B**) Drawing from the photograph demonstrating the length and height of the SH muscle. The approximate location of the postcleithrum is provided as a landmark to connect this drawing with Fig. 3.

A total of five *E. lateralis*, 206 ± 7.9 mm (SEM) standard length, were collected and maintained for use in this study. The number of individuals used in each of the analyses of the study is presented in Table 1. All individuals were obtained by beach seine from Jackson Beach on San Juan Island, WA, and housed in flow-through seawater at the University of Washington’s Friday Harbor Laboratories. All husbandry and experimental procedures on *E. lateralis* were approved by the Institutional Animal Care and Use Committee of the University of Washington. Gross dissection was used to describe the SH muscle in *E. lateralis* (Fig. 1).

**Table 1:** Individual fish used for each type of data collection (numbers represent variable count or sample size with plus or minus symbols [±] denoting whether data were collected for that individual)

	Cameras	External muscle strain (number of trials)	Internal muscle strain (number of trials)	Muscle mass	3D kinematics (number of trials)
*Embiotoca lateralis 02*	3	—	—	−	5
*Embiotoca lateralis 03*	3	10	—	+	8
*Embiotoca lateralis 04*	3	6	—	+	6
*Embiotoca lateralis 06*	2	3	—	+	—
*Embiotoca lateralis 07*	2	3	—	+	—
*Lepomis macrochirus 01*	4^a^	7	7	+	—
*Lepomis macrochirus* ^b^	—	—	—	+	—
*Micropterus salmoides* ^c^	—	—	—	+	—

a Camera count includes two light cameras and two X-ray cameras.

b Camp et al. (2018) two Individuals.

c Camp et al. (2015) three Individuals.

### Marker attachment

**Fig. 2:**
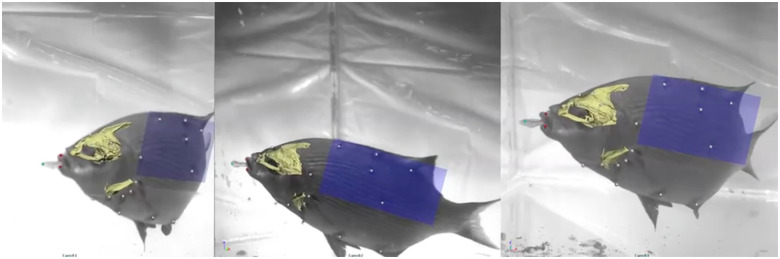
VROMM animation of suction feeding in a striped surfperch. Three camera views with neurocranium, cleithrum, tracked markers (animated as white spheres), and body plane (dark blue) animated from body markers. See Supplementary Movie S1.

**Fig. 3:**
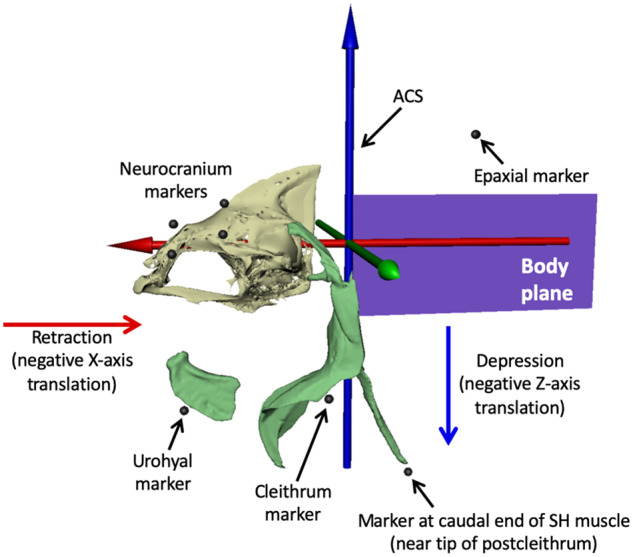
ACS and external marker locations. We animated the body plane from at least five beads attached to the outside of the body (see Fig. 2) and parented the motion of the ACS to the body plane, with the blue *Z-*axis pointing dorsally, the green *Y-*axis pointing laterally to the left, and the red *X-*axis pointing rostrally. Depression of the urohyal and cleithrum was measured as negative translation of their associated markers along the *Z*-axis, and retraction was measured as negative translation along the *X*-axis. SH muscle strain was measured as the change in distance between the urohyal marker and the marker at the caudal end of SH muscle (near the tip of postcleithrum), and epaxial strain from the most caudal neurocranium marker to the most cranial epaxial marker. Mesh models of the urohyal, cleithrum, and postcleithrum are shown for reference. These bones were not animated; their motions were measured from translations of their attached external markers.

White plastic beads (1.5 mm diameter) with holes (i.e., beading hobby beads) were attached to the skin of all individuals in order to track the motions of the skeletal elements and the length changes of the underlying muscles. Fish were anesthetized with a 0.05 g L^−1^ buffered solution of MS-222 and ventilated with a gentle stream of water through a small tube inserted into the mouth. Once the fish were anesthetized, vicryl suture was threaded through the holes in the beads and sutured to the skin just superficial to the bones and muscles of interest: four neurocranium beads, three cleithrum beads, one urohyal bead, one postcleithrum bead, one epaxial muscle bead, and at least five markers to define the 3D position of a body reference plane (Figs. 2 and 3). This marker set enabled tracking the motions of the neurocranium and individual bone beads (one bead each for the urohyal and cleithrum, Fig. 3) relative to a body plane, representing the mean position of the five body markers, as well as measuring length changes of the SH and epaxial muscles. Hypaxial muscle regions in *E. lateralis* were sampled as well, but problems with lighting and the resulting high marker tracking errors led us to exclude the hypaxial muscle data from this study.

### Video recording and camera calibration

Suction feeding events were recorded for each fish using two or three Photron 1024 PCI high-speed video cameras in 30 gal tanks with dimensions 92 cm × 32 cm × 42.5 cm. When operating with two cameras, each was arranged to collect an oblique perspective of the visible plane of the tank. A third optional camera was placed perpendicularly for certain tested individuals, depending on camera availability. Fish were fed non-elusive prey, consisting of pieces of shrimp or squid cut to ∼50% of mouth gape diameter. Calibration images were taken before and after filming for each set of strikes to calibrate the camera positions. Using a 3D calibration object marked with 26 white circles of known relation to one another, XMALab software (Knörlein et al. 2016; bitbucket.org/xromm/xmalab) was used to automatically detect and optimize the internal and external camera parameters for all cameras (10,000 iterations for optimization). The known spacing of calibration circles was also used to account for the effects of image distortion and the refraction of light through water.

Video was captured at a resolution of 1024 × 1024 pixels, at least 500 frames per second, and 1/1000 s shutter speed under high-intensity LED lighting. The video data for this publication have been deposited in the XMAPortal (xmaportal.org) in the study “VROMM Suction Feeding in Fishes” with permanent ID BROWN50 and in the ZMAPortal (zmaportal.org) in the study “Sunfish External Marker Tracking” with permanent ID ZMA2. Video data are stored in accordance with best practices for video data management in organismal biology (Brainerd et al. 2017).

### Marker tracking and VROMM

Following feeding trials, all individuals were euthanized by MS-222 overdose in a 1.0 g L^−1^ buffered solution. Fish were then CT scanned with a Bruker Skyscan 1173 microCT scanner (Bruker, Kontich, Belgium) at a voxel size of 60–140 microns or a Fidex veterinary CT scanner (*Animage*, Pleasanton, CA, USA) at a voxel size of 150 microns. All scans were segmented in the open-source software Horos (Horosproject.org) to isolate and generate mesh surface models of individual bones and markers. Segmented models were cleaned and refined with MeshLab (MeshLab.net) for the removal of non-manifold pieces and CT artifacts. The CT coordinates of marker centroids were then calculated in the animation software Autodesk Maya (Autodesk, San Rafael, CA, USA) using XROMM Maya Tools (https://bitbucket.org/xromm/xromm_mayatools).

The 3D positions of the white plastic markers in the video sequences (Fig. 2) were tracked in XMALab (Knörlein et al. 2016). The 3D coordinates of the markers, together with their CT marker positions, were then grouped by bone into rigid bodies. The combining of individual 3D marker positions into a single grouped rigid body allows XMALab to calculate rigid-body transformations, which reproduce the precise movements of the bones of interest and serve to animate the bone models behind the VROMM process. Rigid body transformations were generated for the neurocranium and an artificially set body plane, which was used as a relative point of reference (Fig. 2). The pose of the body plane in any given frame of video is determined by the best fit for the positions of at least five body markers. Marker-to-marker distances within an identified rigid body, the neurocranium, was also used to determine marker-tracking precision. As the distance between markers that are attached to a rigid body should be constant, the standard deviation (SD) of those distances indicates precision. The mean SD of the six pairwise marker-to-marker distances for the neurocranium markers was 0.17 ± 0.02 mm across all trials used in this study (22 external muscle strain trials plus 5 kinematics trials not used for muscle strain; see Table 1). No significant differences in precision (ANOVA, *F* = 0.0927 *P *=* *0.765) were found between the trials with two versus three cameras.

After calculating rigid body transformations in XMALab, we imported mesh bone models of the neurocranium into Autodesk Maya for animation. Using XROMM Maya Tools, rigid body transformations from XMALab were imported into Maya and used to animate the neurocranium mesh models and body plane. The end product of the VROMM process was a computer-based animated scene (Figs. 2 and 3), ready for the extraction of quantitative data.

### Analysis of VROMM animations and marker motion

Motion data were extracted from VROMM animations with both a joint coordinate system (JCS) and an anatomical coordinate system (ACS). An ACS establishes a 3D coordinate system for measuring the XYZ translations of points in a specific frame of reference and a JCS measures the rotation and translation of two rigid bodies relative to each other (Grood and Suntay 1983). A JCS was placed roughly 10 mm caudal to the head to measure the dorsal rotation (elevation) of the neurocranium relative to the body plane (Jimenez et al. 2018). An ACS was placed and parented to the body plane to decompose translation of the urohyal and cleithrum markers into retraction (negative *X*-axis translation) and depression (negative *Z*-axis translation) as reported by the 3D marker positions of urohyal and cleithrum markers (Fig. 3).

All means are reported with ± standard error. JMP Pro 12.0.1 (SAS Institute Inc.) was used to perform an ANOVA to test for differences among tested individuals in retraction and depression of the urohyal and cleithrum and to look for correlations between retraction and depression of these elements and neurocranial elevation. R Studio was used to calculate 3D marker-to-marker distances exported from XMALab, generate strain and shortening velocity values, and to perform an ANOVA to test for significant differences among individuals in reported values for strain and shortening velocity at peak gape.

### Measurements of muscle strain from external markers

Muscle strain and shortening velocities were determined from measurements of marker-to-marker distances from markers attached to the skin. Markers spanned the full length of the SH muscle and a portion of the epaxial musculature, ranging approximately from the supraoccipital crest to the first dorsal spine (Fig. 3). Gross dissection revealed that the skin overlying the muscles was tightly attached, suggesting that the movements of the musculature could be captured by tracking the motion of the skin superficial to these muscles. Muscle shortening values are reported here as a percentage of initial muscle length prior to the initiation of a feeding strike. Whole-muscle velocity was calculated instantaneously at each time step as the change in normalized muscle length over time, and expressed in initial lengths per second (L_i_s^−1^), with shortening indicated by positive velocities.

Given that this method of data collection was previously untested, evaluation of this external marker tracking was necessary to ensure both accuracy and precision of external marker data. Evaluation of this method occurred post-collection of the *E. lateralis* dataset and was completed in a species of similar body type and degree of skin-to-muscle affixation, *L. macrochirus*, at Brown University. All procedures on *L. macrochirus* were approved by the Brown University Institutional Animal Care and Use Committee.

External marker tracking was compared against the previously validated method of fluoromicrometry (Camp et al. 2016). Internal markers for fluoromicrometry were injected into the SH and epaxial musculature of *L. macrochirus* and tracked according to the methods described by Camp et al. (2016), while external markers (the same size and type of plastic beads used for *E. lateralis*) were sutured to the skin just superficial to the internally placed fluoromicrometry markers in the same individual. Strain and shortening velocity were simultaneously measured by the two methods for seven feeding strikes in one *L. macrochirus* and were then compared to determine both the precision and accuracy of the external tracking method. Comparisons took place by employing linear least squared regressions to compare the data generated by the external marker tracking method as a function of the previously validated fluoromicrometry (same method as Camp et al. 2016). Root mean square error (RMSE) of plotted data was taken as a measure of external marker tracking precision, while the accuracy of the method was expressed as the difference between the slope of the regression line and an idealized slope of 1, which would indicate a perfect match between the two methods.

One of the goals of this paper is to compare SH and epaxial strain in *E. lateralis* with *L. macrochirus* and *M. salmoides*. As fluoromicrometry measurements of epaxial and SH strain were taken here for validation from one *L. macrochirus* individual (*n* = 7 strikes), we combine these newly collected fluoromicrometry data with the previously reported fluoromicrometry results presented by Camp et al. (2018). The combination of muscle strain results from the two studies brings the total number of *L. macrochirus* individuals for which we have SH and epaxial strain from fluoromicrometry to *n* = 3 (Table 1).

## Results

### SH morphology

We used gross dissection to determine the attachment points of the SH muscle in *E. lateralis* (Fig. 1). Rostrally, the SH attached just caudal to the anterior condyle of the urohyal bone, bearing attachments all along the length of the urohyal as the keel of the bone bisected the muscle down its midline; this is typical of the SH muscle in teleost fishes (Winterbottom 1973). We observed that a few fibers of the SH attach directly to the cleithrum. However, a majority of muscle fibers continue past the cleithrum, both laterally and ventrally, with some inserting on the postcleithrum of the pectoral girdle and others attaching to a particularly thick myoseptum of the hypaxial musculature. At these hypaxial attachment points, the muscle fiber angles of the SH differ from the fiber angles of the hypaxial musculature.

### Urohyal and cleithrum kinematics

**Fig. 4:**
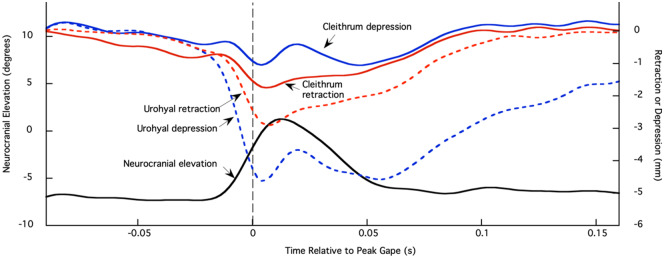
Neurocranial elevation (degrees) and retraction and depression of the urohyal and cleithrum (millimeter) markers from one feeding event. Left axis is neurocranial rotation in degrees relative to the body plane and right axis is retraction or depression (in millimeter) of the urohyal and cleithrum markers (see Fig. 3 for marker placements and ACS). Neurocranium, black; urohyal retraction, red dashed; urohyal depression, blue dashed; cleithrum retraction, red solid; cleithrum depression, blue solid. Time zero and gray dashed line correspond to peak gape.

**Fig. 5:**
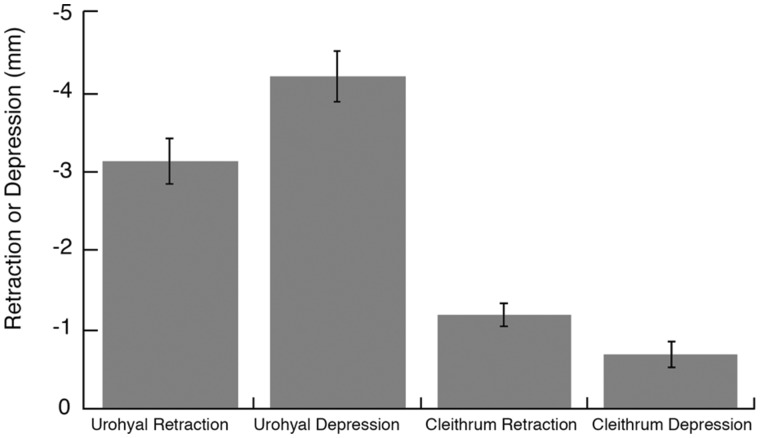
Urohyal and cleithrum kinematics. Mean retraction and depression (±SEM) of the urohyal and cleithrum for 19 strikes from three individual fish. Results from individual fish were not significantly different for all four variables (*P* > 0.05), so results were pooled.

The urohyal and the cleithrum both undergo considerable retraction and depression relative to the body plane during suction feeding strikes of *E. lateralis* (Fig. 4). Results from individual fish were not significantly different for the four cleithrum and urohyal variables (*P *>* *0.05), so results were pooled across 19 strikes from three individuals (Fig. 5). The urohyal marker depressed more than it retracted (*P *=* *0.015), whereas the cleithrum marker retracted more than it depressed (*P *=* *0.028). When comparing relative motions of the two bones, the urohyal both retracted (*P *<* *0.0001) and depressed (*P *<* *0.0001) greater magnitudes than the cleithrum. To determine whether strikes with high cranial elevation also showed large urohyal and cleithrum motions, retraction, and depression of the urohyal and cleithrum were plotted against neurocranial elevation (Supplementary Fig. S1). No correlation was found between the magnitude of any of the motions of these ventral expansive elements and the magnitude of neurocranial elevation (*P *>* *0.85 for all four correlations).

### Muscle strain and shortening velocity

**Fig. 6:**
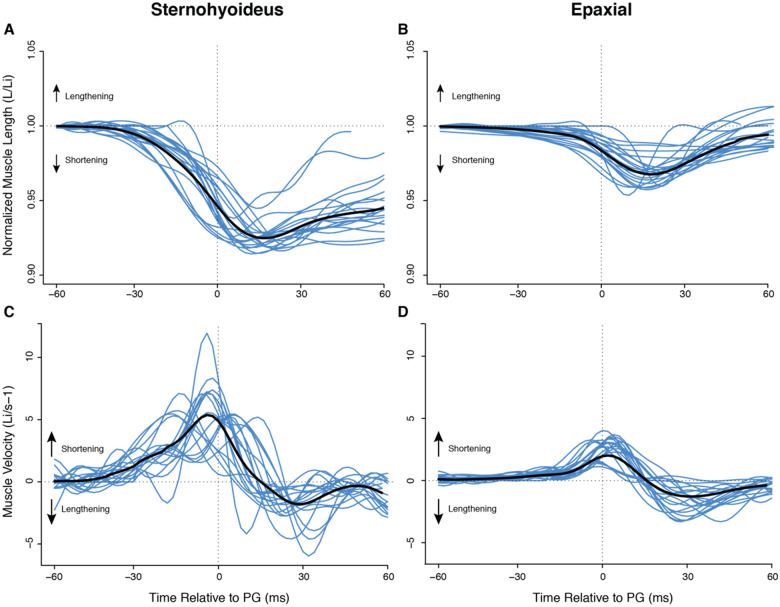
Normalized muscle length and velocity during suction feeding in *E. lateralis*. Time is relative to peak gape. Traces from individual strikes (blue lines) are shown (*n* = 22 strikes from four individuals), with the mean for all strikes in black. (**A**, **B**) Change in muscle length over time normalized by mean initial muscle length (Li) for epaxial and SH, respectively. Decreasing values indicate shortening. (**C**, **D**) Instantaneous velocity for epaxial and SH. For velocity, positive values indicate shortening.

In *E. lateralis*, the epaxial and SH muscles began shortening before peak gape and continued shortening after peak gape in all recorded strikes across all individuals (Fig. 6). Results from individual fish were significantly different for strain and shortening velocities, so results are reported here as the mean of means for non-homogenous data. In the epaxial musculature, peak strain occurred 16 ± 2.4 ms after peak gape, while peak strain in the SH occurred 15 ± 4.0 ms after peak gape. The mean magnitude for all measured epaxial strains was 3.9 ± 0.50%, and the mean SH strain was 7.2 ± 0.55% with measures of strain ranging from 4.2% to 8.4% of initial muscle length. Table 2 presents these data for *E. lateralis* as well as data from previous studies of these variables in largemouth bass and bluegill for comparison.

**Table 2:** Mean magnitudes ±SEM^a^ of relative muscle mass, strain, and shortening velocity at peak gape for striped surfperch, largemouth bass, and bluegill sunfish

	SH % axial muscle mass	SH strain (%)	SH velocity (L_i_/s^−1^)	Epaxial strain (%)	Epaxial velocity (L_i_/s^−1^)
*Embiotoca lateralis*	8.8 ± 0.59	7.2 ± 0.55	4.9 ± 0.65	3.9 ± 0.50	2.7 ± 0.51
*Lepomis macrochirus*	4.0 ± 0.99	12.0 ± 1.0	4.4 ± 0.50	3.9 ± 0.50	2.2 ± 0.30
*Micropterus salmoides*	1.7 ± 0.08	1.3 ± 0.30	0.1 ± 0.09	4.7 ± 0.30	1.0 ± 0.06

a *n* = 4 individuals for *E. lateralis* muscle strain data and *n* = 3 for all other values. *Lepomis macrochirus* and *M. salmoides* data from Camp et al. (2015) and Camp et al. (2018), respectively, with additional SH and epaxial strain and velocity values from fluoromicrometry for a third *L. macrochirus* added here. Axial muscle mass was taken as the total mass of the epaxial and hypaxial musculature following gross dissection.

### Accuracy and precision of external marker tracking for muscle strain

**Fig. 7:**
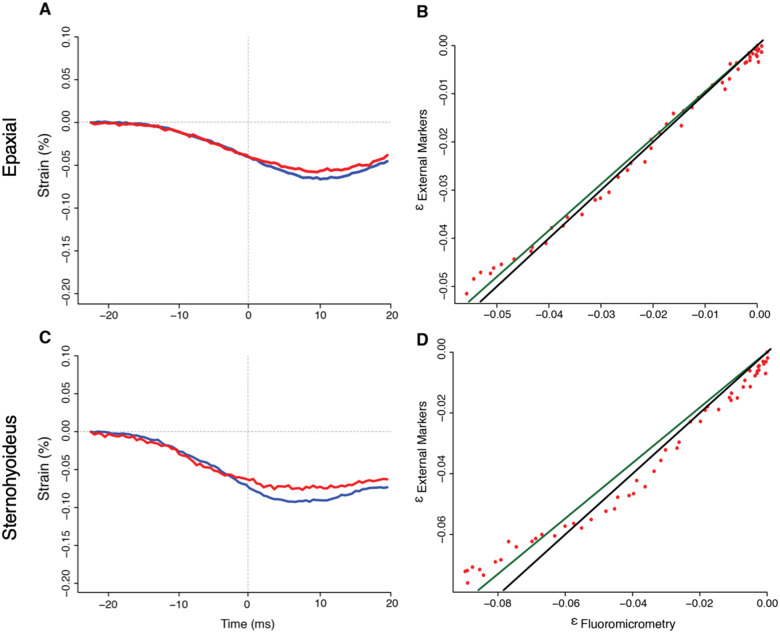
Evaluation of external muscle marker tracking method relative to fluoromicrometry for SH and epaxial muscle length in a bluegill sunfish, *L. macrochirus*, for a representative trial. Muscle strain was measured both by fluoromicrometry (blue) and external marker tracking (red) for (**A**) epaxial and (**C**) SH muscles. Regression plots comparing the generated strain values from both methods are presented for the (**B**) epaxial and (**D**) SH. Strain measurements were compared following methods in Camp et al. (2016). External marker strain is plotted as a function of fluoromicrometry measured strain and fit with a linear regression line (dark green), the slope of which was compared to the 1:1 ratio of the ideal line (black). Red points represent the true strain calculations when plotting εexternal as a function of εfluoromicrometry. ε is a SI unit for the unit-less strain.

Precision and accuracy were calculated for the external marker tracking technique as a test of the validity of using external markers to measure internal muscle strain in fishes, such as *E. lateralis* and *L. macrochirus*, in which the skin is tightly attached to the muscles of interest. Precision and accuracy of external muscle strain measurements were compared with those from internal markers from X-ray imaging (i.e., fluoromicrometry, Camp et al. 2016) in *L. macrochirus* (Fig. 7). Mean epaxial strains at peak gape for this individual were 6.5 ± 0.79%_External_ and 6.9 ± 1.1%_Internal_ while average SH strains at peak gape were 7.37 ± 0.51%_External_ and 8.22 ± 0.33%_Internal_. Mean epaxial velocities at peak gape were 1.27 ± 0.19(L_i_s^−1^)_External_ and 1.45 ± 0.17(L_i_s^−1^)_Internal_ while average SH velocities were 1.64 ± 0.52(L_i_s^−1^)_External_ and 2.48 ± 0.83(L_i_s^−1^)_Internal_.

Precision was taken as the RMSE for all *L. macrochirus* trials: for the epaxial musculature, mean precision was 0.60 ± 0.20% strain, and for the SH, mean precision was 1.06 ± 0.10% strain (*n* = 7 strikes). Accuracy, measured as the mean ratio between the slope of the regression line between the external and internal marker data and the slope of an idealized regression line with a slope of 1, was 0.96 ± 0.03 for the epaxials and 0.90 ± 0.03 for the SH. Hence, the external marker method underestimated true strain values by a margin of 4.0% and 10% for the epaxials and SH, respectively.

## Discussion

Our results indicate that the striped surfperch is a species of fish that is capable of utilizing the bifunctionality of the SH muscle. Evidence of the SH serving primarily as a transmitter of the power generated by the hypaxial musculature, to the hyoid apparatus, is steadily increasing (Van Wassenbergh et al. 2007; Camp et al. 2015; Camp et al. 2018). Surfperch possesses a relatively large SH muscle (Fig. 1;Table 2) and also displays patterns of both skeletal kinematics (Fig. 4) and SH muscle shortening (Fig. 6) that closely align with expected periods of peak suction power production just prior to peak gape. These results match our prediction that we would find SH shortening during suction feeding in *E. lateralis*, supporting the prior results from clariid catfishes and centrarchids that the SH does shorten in species with a relatively large SH, and does not shorten but instead acts like a stiff ligament to transmit hypaxial muscle power in species with a relatively small SH.

### Structure and function of the SH

While generally considered a muscle of the head region, the sites of muscle attachment for the relatively large SH in the striped surfperch extend well past the caudal end of the neurocranium (Fig. 1). In many fishes, the caudal attachment for this muscle is the cleithrum of the pectoral girdle (Winterbottom 1973; Lauder 1980); the extension of the surfperch SH to the postcleithrum enables an increase in the overall muscle mass in *E. lateralis*. The third myocomma of the muscle, thought to differentiate between the SH and the hypaxial musculature (Winterbottom 1973), extends caudally past the operculum, contrary to both the largemouth bass and bluegill condition, increasing SH muscle mass in *E. lateralis*. Relative to axial muscle mass, the SH muscle in *E. lateralis* is more than twice as large as the SH in *L. macrochirus*, and five times the size of the SH in *M. salmoides* (Table 2). The greater relative muscle mass would thus increase the power of hyoid depression, and likely influence suction feeding performance (Aerts 1991; Svanbäck et al. 2002; Van Wassenbergh et al. 2007). However, even in *E. lateralis*, the mass of the SH is only 8.8% of the mass of the axial musculature, suggesting a relatively small, but still likely meaningful, contribution of the SH to overall muscle power.

### Skeletal kinematics

Motions of the urohyal and cleithrum support the inference that SH shortening contributes power to striped surfperch suction feeding, as well as transmitting cleithrum motion to the hyoid apparatus. If actively shortening, the action of the SH is to retract the hyoid apparatus by pulling the urohyal and hyoid bars postero-ventrally (Lauder 1985; Aerts 1991). This active contraction of the SH is demonstrated by the relative degree of urohyal retraction and depression, in comparison to the cleithrum. In the case of largemouth bass, the SH does not consistently shorten during peak power production; hence, the magnitudes of urohyal and cleithrum retraction are similar (Camp et al. 2015). In striped surfperch, our observation of significantly greater retraction and depression of the urohyal relative to the cleithrum (Figs. 4 and 5) suggests that hyoid depression was indeed facilitated by the shortening of the SH rather than retraction of the cleithrum alone.

Additionally, motions of the urohyal, relative to the body plane (Fig. 4), were found to peak during periods of neurocranial elevation. Lifting of the neurocranium results from the shortening of epaxial muscles and serves to increase buccal cavity volume by rotating the neurocranium dorsally. While this occurs, peak postero-ventral motions of the urohyal, relative to the body plane, also take place within the surfperch system (Fig. 4). In combining the depression of the “floor” of the fish mouth with the elevation of the “roof” of the mouth by neurocranial elevation, the potential magnitude of buccal volume change increases in the system (Van Wassenbergh et al. 2015). Thus, it seems reasonable to expect that the magnitude of neurocranial elevation and hyoid depression would be correlated, even when both are measured separately relative to the body of the fish, that is, a higher performance strike should recruit both dorsal and ventral expansion. Surprisingly, neither peak urohyal depression nor peak cleithrum retraction is correlated with neurocranial elevation in *E. lateralis* (Supplementary Fig. S1). A similar lack of correlation was found in *M. salmoides* (Camp and Brainerd 2014; Supplementary Fig. S1), suggesting an interesting area for further research.

### Muscle strain rates and power production

Both the timing and rate of SH muscle shortening further emphasize the likelihood of this muscle assuming the role of a power generator as well as a power transmitter in *E. lateralis*. We consistently observed peak shortening of the SH muscle just following the occurrence of peak gape (Fig. 6), an advantageous situation given that peak suction power has been found to occur just prior to peak gape during suction feeding (Camp et al. 2015; Camp et al. 2018). In order to achieve the observed maximum SH shortening following peak gape, the surfperch system also reaches its maximum observed rate of muscle shortening shortly before peak gape. At an average shortening rate of 4.9 ± 0.65 L_i_/s^−1^, the SH muscle is potentially producing near-optimal levels of muscle power. The timing of this power production is crucial to the process of suction feeding as the high-velocity fluid flows, generated by the efforts of the musculature to expand the buccal cavity, must occur just prior to peak gape to increase the chances of prey capture success (Holzman et al. 2007; Bishop et al. 2008).

Whether optimal power is produced depends in part on how fast the muscle is shortening. The relationship between muscle force, velocity, and power is such that at approximately one-third of maximum muscle velocity (1/3 V_max_), muscle power production reaches a maximum for that particular muscle (Askew and Marsh 1998; Carroll et al. 2009). While this study did not directly measure muscle power, and Vmax for the epaxial and SH muscles of *E. lateralis* are unknown, the comparison of surfperch musculature to the heavily studied *M. salmoides* and *L. macrochirus* systems suggests that both the epaxials and SH in the striped surfperch are effective power producers. Mean shortening velocity at peak gape in the striped surfperch was 4.9 ± 0.65 and 2.65 ± 0.51 Ls^−1^ for the SH and epaxials, respectively. The SH and epaxial values for *L. macrochirus* were similar at 4.4 ± 0.50 and 2.2 ± 0.30 Ls^−1^. *Lepomis macrochirus* was found to be generating high mass-specific muscle power for suction feeding (Camp et al. 2018), suggesting that the muscle velocities in *E. lateralis* may also generate near-optimal power. By contrast, muscle velocities in *M. salmoides* were found to be lower, at nearly zero for the SH and 1.0 ± 0.063 for the epaxials, which is consistent with the SH generating no power and the epaxials generating less than their maximum potential, even in the highest-power strikes (Camp et al. 2015; Jimenez and Brainerd 2020). Hence, the similarity of SH velocity in *L. macrochirus* and our study species provides further evidence that the SH contributes power to suction feeding in *E. lateralis*.

### External marker tracking for measuring muscle strain

We present 3D external marker tracking as a method useful in certain specific conditions, as opposed to a universally applicable technique for measuring muscle strain. The power of the method lies in its ability to measure internal muscle strain using external observations and minimal resources, relative to fluoromicrometry and sonomicrometry. However, external marker tracking only works if the skin is tightly connected to the underlying muscles, as is the case for the epaxial, hypaxial, and SH muscles of some fishes. We emphasize that the results here are from two to three cameras and 3D motion tracking, and caution that 2 D external marker tracking is unlikely to be suitable for this application, given that the measured 2 D distance between markers varies substantially if the fish are not precisely lateral to the camera or changes distance from the camera.

We tested this 3D method in bluegill sunfish by comparing the results from external markers with internal markers measured with fluoromicrometry (Camp et al. 2016). Bluegill sunfish were used for these validation experiments, rather than striped surfperch, due to the availability of bluegill at Brown University, where the X-ray machines are located. We found that external markers underestimated true muscle strain by 4% in the epaxials and 10% in the SH. The skin overlying fish muscle is comprised of wound collagenous fibers (Nadol et al. 1969; Brown and Wellings 1970; Hawkes 1974), which vary in their degree of attachment depending on species and location on the body. Therefore, how tightly attached overlying skin is to underlaying muscle likely determines how much influence deep muscular layers have on the superficial skin layers, and the reverse, as tightly attached skin has been thought to have some influence on muscle force (Videler 2012). For this study, we likely underestimated true strains and strain rates in the striped surfperch, particularly for the SH. Since our main conclusion is that the SH shortens with a high velocity, the underestimate makes our conclusion more conservative.

We propose that 3D external marker tracking can be an effective way to determine whether the epaxial, hypaxial, and SH are shortening during suction feeding in some fishes, but is likely to underestimate true strains to a varying degree depending on how tightly the skin is attached to the muscles. In this study, we did not measure or validate hypaxial strain directly due to problems with how we set up the lighting. For both *E. lateralis* and *L. macrochirus*, the lighting was at an oblique-dorsal angle, which resulted in the poor lighting of the ventral side of the fish. This poor lighting introduced so much noise into the 3D marker tracking that hypaxial strains were obscured. Nonetheless, with good lighting, external marker tracking for hypaxial musculature should have similar accuracy and precision as our results for epaxial musculature. In addition, this 3D external marker method might also be useful for measuring skin strain *in vivo*. When possible, fluoromicrometry and sonomicrometry remain more accurate and precise methods for measuring muscle strain (Camp et al. 2016).

## Concluding remarks

Numerous electromyographic studies have shown that the SH is electrically active during the process of suction feeding (Lauder 1980; Grubich 2001; Westneat 2005). The active SH muscle has been generally assumed to shorten in response to this electrical activity and therefore contribute to the overall suction power. However, the possibility of isometric muscle activity occurring with no contribution to suction power requires us to further investigate whether the SH muscle actually shortens in response to the observed nervous stimulation. In this study, we tested the idea that the relatively large size of the *E. lateralis* SH is indicative of the muscle’s ability to actively shorten and contribute power to the suction feeding strike. We found that the SH did indeed shorten in all recorded feeding strikes, supporting the emerging hypothesis that relative SH size may predict whether the SH acts bifunctionally to generate and transmit power or isometrically with the sole function of transmitting hypaxial muscle power.

## Supplementary Material

obaa021_Supplementary_Data
